# Experimental Assessment of Stress–Strain Response in Filament-Wound GFRP Pipes Under Internal Pressure Loading

**DOI:** 10.3390/ma19030639

**Published:** 2026-02-06

**Authors:** Costin Nicolae Ilincă, Ibrahim Naim Ramadan, Rami Doukeh, Adrian Neacsa, Alin Diniță, Eugen Victor Laudacescu, Marius Gabriel Petrescu, Marius Bădicioiu, Ștefan Alexandru Gavrilă

**Affiliations:** Mechanical Engineering Department, Petroleum-Gas University of Ploiesti, 100680 Ploiesti, Romania; icostin@upg-ploiesti.ro (C.N.I.); rami.doukeh@upg-ploiesti.ro (R.D.); adinita@upg-ploiesti.ro (A.D.); pmarius@upg-ploiesti.ro (M.G.P.); mbadicioiu@upg-ploiesti.ro (M.B.);

**Keywords:** FRP piping systems, design standards, hoop stress, strain gauges

## Abstract

Fiber-reinforced polymer (FRP) pipes are increasingly used in pressure piping systems due to their corrosion resistance and favorable mechanical performance; however, the direct experimental validation of design assumptions adopted in international standards remains limited. The objective of this study is to experimentally validate the mechanical response and stress distribution of filament-wound GFRP pipes under representative loading conditions and to assess the consistency of the measured behavior with the allowable-stress design framework of ISO 14692 and complementary ASME and BS codes. In this study, the mechanical behavior of filament-wound glass fiber-reinforced polymer (GFRP) pipes is investigated through a combined experimental program including tensile, bending, and full-scale internal pressure tests. Electrical resistance strain gauges were applied in axial and circumferential directions to directly measure deformation under internal pressure up to 31 bar, allowing experimental stresses to be derived using orthotropic laminate relationships. The results demonstrate a predominantly linear elastic response within the service range, followed by progressive damage initiation at higher load levels, with circumferential stresses consistently exceeding axial stresses, confirming a hoop-dominated response. At the maximum applied pressure of 31 bar, axial and circumferential strains reached approximately ε_a_ ≈ 1.30 × 10^−3^ and ε_h_ ≈ 1.60 × 10^−3^, corresponding to experimentally derived stresses of σ_a_^exp^ ≈ 15.3 MPa and σ_h_^exp^ ≈ 18.8 MPa, without catastrophic failure. The novelty of this work lies in the direct integration of full-scale strain gauge measurements with standardized allowable-stress design assumptions, enabling an experimental validation of ISO 14692 that is rarely addressed in existing studies. The experimentally derived stress–strain data show good agreement with theoretical models and provide a direct link between measured behavior and the allowable stress philosophy and design equations defined in ISO 14692 and complementary ASME and BS design codes. The findings validate the applicability of standardized design approaches and provide experimentally grounded support for engineering design decisions in FRP piping systems.

## 1. Introduction

Fiber-reinforced polymer (FRP) piping systems have been increasingly adopted in a wide range of industrial applications, including oil and gas production, chemical processing, water transmission, and offshore installations [1,2], due to their high corrosion resistance [3], favorable strength-to-weight ratio, and reduced maintenance requirements compared with conventional metallic pipelines [4,5]. In aggressive environments, where steel pipelines are prone to corrosion, scaling, or fatigue damage, FRP pipes provide a technically attractive alternative, offering extended service life and improved operational reliability [6]. Among the various manufacturing techniques, filament winding is widely employed for the production of FRP pipes, as it enables controlled fiber orientation and laminate architecture tailored to resist internal pressure and combined mechanical loading conditions [7].

Glass fiber-reinforced polymer (GFRP) composites consist of continuous or discontinuous glass fibers embedded in a thermosetting polymer matrix, forming lightweight structural materials characterized by high specific strength, corrosion resistance, and good fatigue performance [8]. Filament-wound GFRP pipes can be produced using several composite manufacturing routes, ranging from open-mold techniques such as hand lay-up and spray lay-up [9,10,11,12] to closed-mold processes including resin transfer molding (RTM) and vacuum-assisted RTM (VARTM), which improve impregnation quality and reduce void content [13]. For pressure piping applications, filament winding represents the most widely adopted methodology, as it enables automated production with controlled fiber placement (hoop and helical winding patterns), repeatable laminate thickness, and consistent mechanical performance [14]. In addition, pultrusion is employed for constant cross-section FRP profiles and selected tubular components [15], providing high productivity and favorable axial fiber alignment, while matched-die molding methods are typically applied for net-shape parts rather than full-length pressure pipes [16].

With respect to polymer matrices suitable for composite pipes, the literature reports several widely used systems depending on performance requirements and service conditions. Thermoplastic matrices include polyethylene (PE), covering Low-Density Polyethylene (LDPE), Medium-Density Polyethylene (MDPE), High-Density Polyethylene (HDPE), and Linear Low-Density Polyethylene (LLDPE), as well as polypropylene (PP) and polystyrene (PS), which are commonly employed in fiber-reinforced composites due to their processability and cost-effectiveness [5,17,18]. Polyethylene terephthalate (PET), including recycled PET-derived polyester systems, and polycarbonate (PC) have also been investigated as matrix materials offering enhanced thermal or mechanical performance in specific composite configurations [18]. For higher-performance structural and pressure-bearing applications, epoxy-based matrices are frequently adopted due to their superior fiber–matrix adhesion, stiffness, and mechanical reliability, making them particularly suitable for demanding composite pipe systems [1,8,18]. Recent studies have further demonstrated that manufacturing techniques such as prepreg lay-up, vacuum-assisted resin transfer molding, and co-curing play a critical role in controlling interlaminar bonding quality, void content, and residual stresses in glass fiber reinforced epoxy composites. Mechanical and structural analyses indicate that the flexural response, strain distribution, and failure mechanisms of these systems are governed by interlaminar damage initiation and matrix–fiber interaction, with good agreement observed between experimental results and numerical predictions [19,20,21].

The mechanical performance of filament-wound GFRP pipes is strongly affected by manufacturing parameters such as winding angle and fiber volume fraction. Hydrostatic pressure tests on pipes with winding angles between ±40° and ±70° and wall thicknesses of 3.78–5.1 mm showed that optimal pressure resistance and minimal deformation (~0.37 mm) were achieved near ±55° [22], while variations in fiber content (50–70%) were reported to contribute up to ~34% to mechanical degradation and damage evolution [23].

The winding angle governs the balance between axial and hoop stiffness and strength [24], while fiber content and impregnation quality control stiffness, damage initiation, and failure mechanisms [23]. In addition, winding tension and curing conditions influence residual stresses and interlaminar bonding, highlighting the need for controlled processing to ensure reliable mechanical behavior of GFRP pressure pipes [25].

From a mechanical standpoint, and consistent with the influence of manufacturing parameters discussed above, the structural performance of filament-wound glass fiber-reinforced polymer (GFRP) pipes is primarily governed by laminate architecture and fiber orientation. Experimental investigations on GFRP pipes indicate that damage initiation and failure under transverse and pressure-related loading are primarily controlled by interlaminar mechanisms and geometric configuration. Delamination was observed at diametric deflections of about 27–31%, with reaction forces of approximately 1225–1242 N at 5% deflection, confirming the dominant role of interlaminar damage mechanisms in composite pipeline performance [26].

Saghir et al. [27] investigated the tensile behavior of filament-wound GFRP pipes and reported a clear dominance of hoop strength over axial strength due to circumferential fiber orientation. This hoop-dominated response is consistent with classical thin-walled cylinder behavior under internal pressure. However, their study does not link tensile properties to strain gauge measurements under internal pressure nor to the ISO 14692-3 [28] allowable stress design approach.

Recent studies on filament-wound GFRP pipes have further shown that circumferential (hoop) tensile strength governs mechanical performance and durability under aggressive service conditions. Accelerated aging leads to progressive microstructural damage and a reduction in hoop tensile strength, with reported strength retention decreasing to about 75% after long-term exposure, confirming the critical role of hoop-dominated behavior in FRP pipe assessment [29].

In addition to environmental effects, manufacturing parameters play a decisive role in defining the pressure-related mechanical response of composite pipelines. Experimental results on filament-wound GFRP pipes indicate that the winding angle has a dominant effect on pressure-related mechanical properties, with hoop tensile strength exceeding 800 MPa for circumferential fiber orientations, while transverse compressive strength decreases by approximately 50% at low winding angles. These findings confirm that pressure resistance in composite pipelines is primarily governed by fiber orientation [30].

Experimental studies on GFRP pipes under transverse compressive loading further demonstrate that pipe stiffness and failure are strongly influenced by large deformations, with reaction forces of about 1225 N at 5% diametric deflection and failure occurring at deflections above 30%. These results confirm that accurate mechanical characterization of composite pipelines requires accounting for geometric nonlinearity rather than relying on simplified analytical models [31].

Finally, bending-related investigations have shown that structural reinforcement strategies can significantly modify the mechanical response of composite pipelines. Experimental bending tests demonstrated that CFRP-reinforced composite pipes exhibit enhanced mechanical performance, with peak bending loads increasing from 36.02 kN for unreinforced pipes to 40.41 kN and 42.79 kN for pipes reinforced with two and four CFRP layers, respectively, while increasing the winding angle from 0° to 90° led to a clear reduction in load-bearing capacity [32].

Although many studies have examined the mechanical behavior of GFRP pipes using coupon-level tests, bending, or transverse loading, direct experimental validation of design assumptions based on full-scale strain measurements under internal pressure remains limited. In particular, the direct linkage between experimentally derived stresses and the allowable-stress philosophy adopted in ISO 14692 and related design codes is rarely addressed. This study addresses this gap by combining tensile, bending, and full-scale internal pressure tests with axial and circumferential strain gauge measurements, enabling direct stress evaluation and validation of standardized design assumptions for filament-wound GFRP pressure pipes.

## 2. Materials and Specimens

The material investigated in this study is a fiber-reinforced polymer (GFRP) composite produced by Future Pipe Industries (Dubai, United Arab Emirates)**,** consisting of a thermosetting polymer matrix based on polyester, epoxy, or vinyl ester/polyester systems and reinforced with continuous E-glass fibers. The fibers are predominantly arranged in bidirectional, circumferential (hoop), and helical orientations, which is characteristic of the filament-winding manufacturing technology commonly employed for FRP pipeline systems. The average fiber volume fraction was approximately 55–60%, while the resin content accounted for approximately 40–45% of the composite volume. The manufacturing process involved automated filament winding, followed by controlled curing under elevated temperature conditions. This laminate architecture, composed of alternating hoop and helical reinforcement layers, is optimized for internal pressure resistance and is representative of industrial piping applications subjected to complex mechanical loading conditions arising from internal pressure, axial loads, and temperature variations.

**Figure 1:**
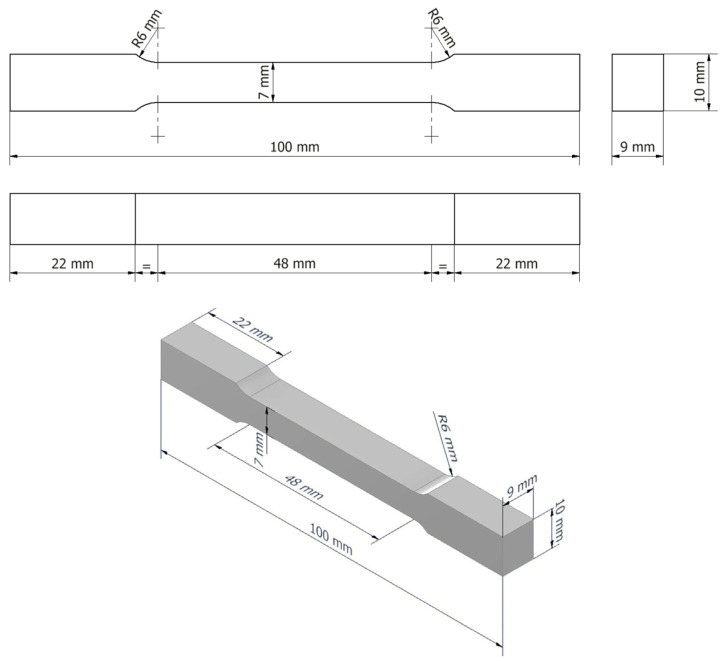
Two-dimensional (2D) drawing of the test specimen geometry (R indicates the radius).

The experimental program was carried out using FRP plates, panels, and filament-wound pipe sections, from which the specimens required for mechanical testing were extracted. Tensile test specimens were prepared according to a standardized geometry, schematically illustrated by the 2D drawing shown in Figure 1, ensuring result reproducibility and comparability with data reported in the literature [33]. Bending test specimens were extracted from the same type of FRP laminate in order to eliminate the influence of structural variability on the mechanical response.

In addition to tests performed on flat specimens, FRP pipe segments were employed for internal pressure testing, conducted in accordance with standardized methodologies, with the aim of evaluating the circumferential (hoop) and axial stresses developed within the pipe wall. Furthermore, strain gauges were applied to selected pipe samples, enabling the monitoring of deformation as a function of internal pressure over the range of 0–31 bar and allowing correlation with theoretical design and calculation models.

All specimens were cut using a CNC water-jet cutting system manufactured by WUXI YC Industry Co., Ltd. (Wuxi, China) (Supplementary Information File—Figure S1), ensuring high dimensional accuracy and avoiding thermal damage to the composite material. This cutting technique also produced clean edges without degraded zones or processing-induced microcracks. Such an approach was required for ensuring the reliability of the experimental results and for minimizing error sources associated with specimen preparation. Figure S2 in the supplementary Information File illustrates the FRP pipe segment before cutting and the corresponding specimen after water-jet machining, highlighting the quality of the cut surfaces and the absence of thermally affected zones.

### 2.1. Normative Framework and Design Criteria

The experimental investigation and data interpretation in this study were conducted within a well-defined normative framework, based on internationally recognized standards and design codes for fiber-reinforced polymer (FRP) piping systems. The primary reference for design and structural assessment was ISO 14692 [28], petroleum and natural gas industries’ glass-reinforced plastic (GRP) piping, which provides the fundamental design philosophy for FRP pipelines, including wall thickness determination, allowable stress limits, safety factors, and classification of loading cases such as internal pressure, external pressure, self-weight, hydraulic loads, and occasional loads.

The determination of circumferential tensile strength and ring stiffness of GRP pipes was based on standardized test methods defined in ISO 8521, ISO 14828, and EN 1394 [34,35], which specify multiple testing approaches (Methods A and B–F) depending on pipe geometry, winding angle, and testing objectives. These standards provide the experimental basis for evaluating hoop stress resistance and deformation behavior under internal pressure or equivalent loading configurations.

Design criteria and verification approaches for non-metallic piping were further supported by ASME B31.3 [36], from which sections dedicated to non-metallic materials were employed, including adapted formulations for wall thickness calculation and criteria for circumferential and longitudinal stresses induced by internal pressure. In addition, ASME RTP-1 [37] was used to complement the analysis, particularly for pressure-loaded FRP components, providing design relationships for wall thickness under internal and external pressure and stability criteria under external pressure conditions.

Finally, BS 7159 [38] was employed as a reference code of practice, from which wall thickness formulations based on allowable strain criteria were extracted. These formulations were used to verify external pressure resistance and ring stability of the laminated FRP pipes.

Based on this normative framework, the standards were jointly employed to define the calculation relationships for wall thickness parameters (t, t_r,min_, t_d_), to establish elastic moduli in axial and circumferential directions, and to derive allowable stress limits from long-term test data and standardized nomograms.

### 2.2. Mechanical Testing

The mechanical characterization of the FRP specimens was carried out through a comprehensive experimental program comprising tensile, bending, and internal pressure tests, designed to evaluate the structural response of the material under loading conditions representative of service conditions in piping systems. Tensile tests were performed using a Walter + Bai LFV universal testing machine, manufactured by Walter + Bai AG Prüfmaschinen (Testing Machines), Löhningen, Schaffhausen, Switzerland, under monotonically increasing axial loading. A total of six tensile tests were conducted under controlled conditions, ensuring a constant strain rate, accurate axial alignment of the specimens, and precise measurement of force and displacement. From the recorded force–displacement and corresponding stress–strain curves, the initial linear elastic region was identified, and the elastic modulus E was determined from the slope of the stress–strain curve in the elastic domain, expressed as E=tanβ, where β represents the inclination angle of the curve within the linear elastic range. Figure S3 in the Supplementary Information file presents representative FRP tensile specimens and the experimental setup used for tensile testing, illustrating the specimen geometry and the loading configuration during the test.

Bending tests were carried out using a dedicated bending test fixture mounted on the same universal testing system, configured for three-point or four-point loading depending on the specimen geometry. This setup ensured a controlled distribution of the bending moment along the specimen span and allowed the evaluation of bending stiffness and equivalent flexural modulus. Six bending tests were performed, yielding force–displacement curves from which the sectional stiffness was determined. In addition, the bending tests enabled qualitative and quantitative assessment of damage initiation and failure mechanisms, including brittle fracture, fiber–matrix decohesion, and interlaminar delamination. Figure S4 in the Supplementary Information file illustrates representative FRP specimens subjected to bending and the corresponding experimental configuration, highlighting the loading arrangement and typical deformation and failure modes observed during the tests.

The resistance to circumferential tensile stress and the behavior under internal pressure were evaluated using internal pressure tests conducted in accordance with the ISO 8521 methodology, following the principles outlined in EN 1394 [34]. Pressurization tests up to failure (Method A) were performed on sealed FRP pipe segments instrumented with strain gauges, allowing direct measurement of deformation during pressurization. Internal pressure was applied until rupture, and the corresponding burst pressure was recorded, from which the maximum associated hoop stress was calculated. Although ISO 8521 also defines ring- and strip-based tensile test methods (Methods B–F), suitable for pipes with different winding angles, the present study focuses primarily on full-scale internal pressure testing, as it provides a more realistic representation of in-service loading conditions. Strain gauges were installed in both axial and circumferential directions, and strain evolution was monitored at internal pressure levels of 0, 15, 20, 26, and 31 bar. Data acquisition and strain monitoring were performed using a dedicated pressure and strain measurement system (Strain gauge amplifier–Quantum X MX1615B/MX1616B-HBK Hottinger Brüel & Kjær-Darmstadt, Germany), enabling direct correlation between applied pressure, measured strains, and theoretical stress predictions used in design calculations. Figure S5 in the Supplementary Information file illustrates the experimental setup for internal pressure testing, including the FRP pipe segment instrumented with strain gauges, the pressurization assembly, and the schematic representation of strain gauge placement along the pipe wall.

### 2.3. Electrical Strain Gauging and Deformation Measurement

Electrical resistance strain gauges were applied to the external surface of the FRP pipe in order to monitor the deformation response under internal pressure loading. The strain gauges consisted of a polymeric backing substrate with a fine-wire resistive grid, specifically designed for surface-mounted measurements on composite materials. The gauges were characterized by a calibrated gauge length I_0_ and width B, selected to ensure accurate strain measurement within the region of interest on the pipe wall.

The strain gauges were connected to a dedicated strain measurement system, forming Wheatstone bridge circuits for the determination of axial strain $\varepsilon_{a}$ and circumferential (hoop) strain $\varepsilon_{h}$. This configuration enabled continuous monitoring of strain evolution during pressurization and ensured reliable acquisition of deformation data corresponding to different internal pressure levels.

The measured electrical signals were converted into axial and circumferential strains according to the following Equations (1) and (2) [39]:

(1) $$\begin{array}{cccc} & \varepsilon_{a}=(I_{a}-I_{0})\cdot 10^{-6} & & \end{array}$$

(2) $$\begin{array}{cccc} & \varepsilon_{h}=(I_{h}-I_{0})\cdot 10^{-6} & & \end{array}$$

Based on the strain–stress relationships for orthotropic FRP laminates under plane stress conditions, the experimentally derived axial and circumferential stresses were calculated using the following Equations (3) and (4) [28,40]:

(3) $$\begin{array}{cccc} & \sigma_{a}^{exp}=\frac{E_{L}}{1-\mu^{2}}\;\left(\varepsilon_{a}+\mu \;\varepsilon_{h}\right) & & \end{array}$$

(4) $$\begin{array}{cccc} & \sigma_{h}^{exp}=\frac{E_{C}}{1-\mu^{2}}\;(\varepsilon_{h}+\mu \;\varepsilon_{a}) & & \end{array}$$

In Equations (1)–(4), $\varepsilon_{a}$ and $\varepsilon_{h}$ denote the axial and circumferential (hoop) strains, respectively, obtained from the corresponding strain gauge signals, where *I_a_* and *I_h_* represent the measured electrical outputs and I_0_ is the initial reference signal at zero load. The experimentally derived axial and circumferential stresses are denoted by $\sigma_{a}^{exp}$ and $\sigma_{h}^{exp}$, respectively. *E_L_* and *E_C_* represent the longitudinal and circumferential elastic moduli of the filament-wound FRP laminate, while μ is the laminate Poisson’s ratio. The elastic constants were determined experimentally and/or adopted in accordance with ISO 14692 recommendations for GRP/FRP piping systems.

Based on these expressions and on the constitutive relationships linking elastic moduli and Poisson’s ratios, as defined in ISO 14692, the axial stress $\sigma_{a}$ and circumferential stress $\sigma_{h}$ associated with the applied internal pressure levels were determined. These calculated stress values were subsequently used for comparison with theoretical design predictions and allowable stress limits specified by the relevant standards.

### 2.4. Data Processing and Determination of Allowable Stresses

The experimental data obtained from mechanical testing and strain measurements were processed following a structured methodology aimed at deriving material properties and design-relevant parameters for FRP piping systems. The first step consisted of determining the elastic moduli of the material in the principal directions. The axial elastic modulus was obtained from tensile test results, based on the axial strain–stress response, while the circumferential elastic modulus was evaluated either directly from strain measurements or indirectly through its relationship with Poisson’s ratio.

The transverse shear modulus G was subsequently determined using standardized relationships defined in ISO 14692, expressed as functions of the axial and circumferential elastic moduli and the corresponding Poisson’s coefficients. These material properties formed the basis for further structural calculations and design verifications.

Wall thickness calculations were then performed for different loading scenarios. For internal pressure loading, wall thickness requirements were evaluated in accordance with ISO 14692, ASME B31.3, ASME RTP-1, and BS 7159. In addition, wall thickness verification under external pressure and ring stability conditions was carried out based on the criteria specified in ASME RTP-1 and BS 7159.

The determination of allowable stresses was conducted through the construction of allowable stress nomograms in accordance with ISO 14692. Characteristic points on the nomogram were obtained from experimental and analytical stress states corresponding to pure internal pressure loading (stress ratio 2:1), combined axial tension and internal pressure loading (stress ratios 1:1 and 2:1), pure axial tension, and pure axial compression. Reduction factors accounting for service life, environmental conditions, maintenance level, and type of loading were subsequently applied in order to derive the design allowable stresses for the FRP piping elements.

## 3. Results and Discussion

### 3.1. Tensile Behavior

**Figure 2:**
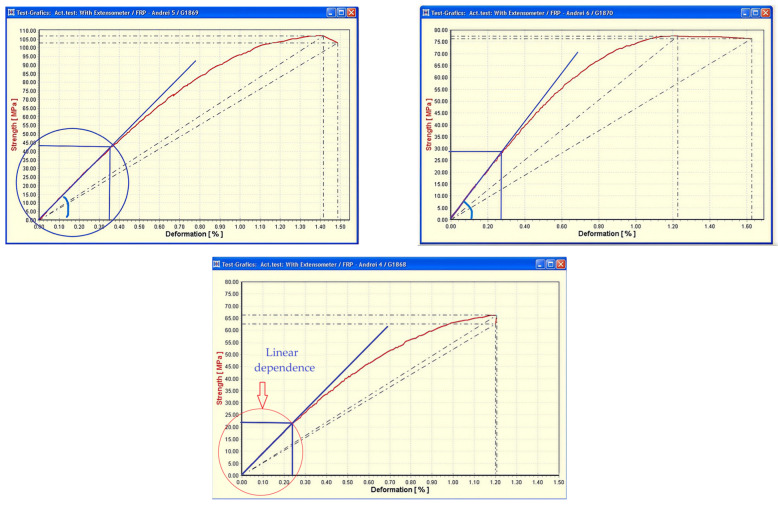
Representative tensile stress–strain curves of filament-wound GFRP specimens, highlighting the initial linear elastic region used for the determination of the longitudinal elastic modulus. The deviation from linearity indicates the onset of matrix cracking and progressive damage development.

The tensile stress–strain curves (Figure 2) exhibit a predominantly linear elastic response at low strain levels, followed by a gradual deviation from linearity as deformation increases. The initial linear region, clearly identified in all tested specimens, corresponds to elastic load transfer governed by the glass fibers and an intact fiber–matrix interface. The slope of this region was used to determine the longitudinal elastic modulus, a key parameter for structural assessment and design of GFRP pipes under service loading conditions. Figure 2 presents representative tensile stress–strain curves selected from three specimens out of a total of six tensile tests performed under identical conditions. The remaining specimens exhibited comparable stress–strain trends and linear elastic behavior, as reflected by the elastic modulus and strength values summarized in Table 1, confirming the repeatability of the tensile response and the reliability of the experimental results.

The tensile specimens reported in Table 1 were nominally identical samples extracted from the same filament-wound GFRP laminate. The observed scatter in mechanical properties reflects the inherent material heterogeneity of composite laminates, associated with local variations in fiber distribution and resin content, rather than systematic differences between specimen types.

As reported in Table 1, the experimentally determined longitudinal elastic modulus values range between approximately 9000 and 12,500 MPa. These values fall within the lower-to-medium stiffness range commonly reported for structural glass fiber–reinforced polymer (GFRP) laminates with non-unidirectional or quasi-isotropic reinforcement architectures, as documented in the literature and design standards [ISO 14692-3] [6,28,41,42]. The tensile specimens reported in Table 1 were nominally identical samples extracted from the same filament-wound GFRP laminate. The observed scatter in elastic modulus and other mechanical properties reflects the inherent variability of composite materials, which can be attributed to local variations in fiber volume fraction, winding angle, and resin distribution, rather than to systematic differences between specimen types, while still indicating a satisfactory level of manufacturing reproducibility [40].

Beyond a characteristic strain threshold, typically between 0.23% and 0.35%, the stress–strain response departs from linearity, signaling the onset of damage mechanisms within the composite structure. This transition is associated with matrix microcracking, followed by localized fiber–matrix debonding and, in some cases, the initiation of interlaminar damage. The stresses corresponding to the end of the linear elastic regime, ranging from approximately 22 to 43 MPa (Table 1), define the upper limit of undamaged material behavior and are of particular relevance for design considerations.

Ultimate tensile strength values obtained experimentally range from approximately 66 to 107 MPa, with corresponding strain at failure between 1.18% and 1.42%.

Failure generally occurs in a relatively abrupt manner, characteristic of brittle fiber-reinforced polymer composites, and is marked by a sudden drop in stress once the maximum load-carrying capacity is reached, as commonly reported for GFRP materials under tensile loading [41,43,44]. However, the presence of a limited non-linear region preceding rupture indicates a progressive accumulation of matrix-dominated damage prior to final fiber fracture.

From the perspective of the allowable stress philosophy defined in ISO 14692, the experimental results provide a consistent basis for the selection of design stresses. ISO 14692 requires that operational stresses in GRP piping systems remain within the linear elastic domain and sufficiently below damage initiation thresholds, ensuring long-term structural integrity under sustained loading conditions. The ratio between the stress level corresponding to linear elastic behavior and the ultimate tensile strength observed in this study indicates a substantial safety margin, supporting the applicability of the investigated laminate for pressure piping applications designed according to this standard.

The moderate variability observed among specimens, as reflected by the dispersion of elastic modulus, linear-limit stress, and ultimate strength values in Table 1, confirms a reasonable degree of material homogeneity and good reproducibility of the filament-winding manufacturing process. Overall, the tensile test results demonstrate a stable and predictable mechanical response, characterized by linear elastic behavior within the service strain range, followed by controlled damage evolution and a predominantly brittle final failure.

### 3.2. Bending Behavior and Load–Displacement Response

**Figure 3:**
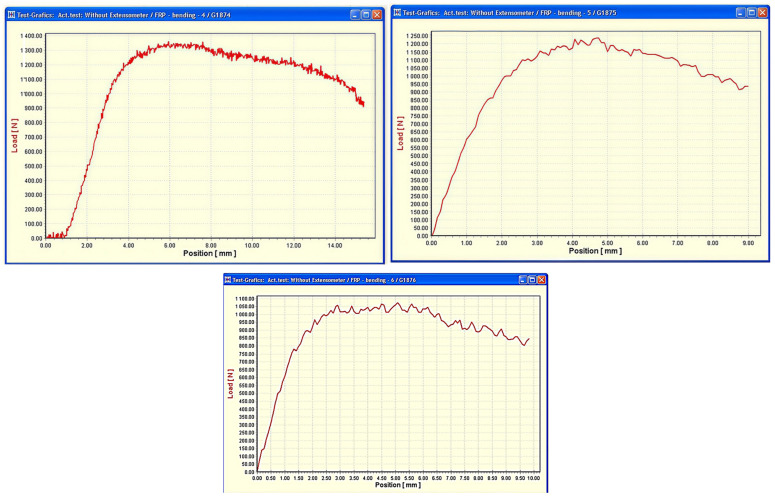
Representative load–displacement curves from three-point bending tests performed on nominally identical filament-wound GFRP specimens under identical experimental conditions. The curves are shown as separate plots due to data acquisition constraints, while their similarity confirms good repeatability of the bending response.

In the initial stage of loading, the curves display a high initial stiffness, characterized by a steep and nearly linear increase in load with increasing displacement (Figure 3). This initial linear segment corresponds to the elastic bending response of the GFRP specimens, indicating an efficient load transfer mechanism within the composite section, dominated by the elastic response of the fibers and an intact fiber–matrix interface. The nearly identical slopes observed in this region for all three curves confirm a consistent bending stiffness and good material homogeneity among the tested specimens. Figure 3 presents load–displacement curves obtained from three representative specimens selected from a total of six bending tests performed under identical three-point bending conditions on nominally identical GFRP specimens. Due to the data acquisition format of the testing system, the curves are presented as separate plots within the same figure; however, their close similarity allows direct comparison and confirms the repeatability of the bending response.

As the applied displacement increases, the load–displacement response remains approximately linear up to a well-defined critical displacement, beyond which a gradual deviation from linearity becomes evident. This deviation marks the onset of matrix cracking and interlaminar damage in the tensile zone of the bent specimens, where tensile stresses locally exceed the matrix-dominated strength limit. Following crack initiation, a progressive stiffness degradation is observed, reflected by the reduced slope of the curves and the development of a nonlinear response.

The post-cracking behavior is characterized by a gradual evolution toward failure rather than an abrupt collapse. The curves exhibit a peak load followed by a softening branch, indicating a limited ability of the composite to redistribute stresses after damage initiation. The experimental data show that maximum applied loads are reached at displacement levels ranging from approximately 2.5 to 6 mm, with corresponding peak forces between about 1000 and 1400 N, as presented in Table 2. The dispersion of peak load values reflects the inherent variability associated with composite materials and local differences in fiber architecture or interlaminar bonding.

The observed failure modes further support the interpretation of the mechanical response. Failure predominantly occurs in the tensile region of the specimens and is associated with fiber rupture accompanied by fiber–matrix decohesion. In addition, interlaminar delamination between reinforcement layers was observed, particularly in regions subjected to stress concentrations (Figure S4). These damage mechanisms are consistent with the gradual stiffness degradation and the development of a non-linear mechanical response following crack initiation, a behavior that has been widely reported for glass fiber–reinforced polymer composites under tensile and flexural loading [6,45,46,47].

Overall, the bending test results confirm that the studied FRP elements exhibit a good capacity to sustain bending moments, as evidenced by the relatively high peak loads attained prior to failure. However, the apparent ductility remains limited, as indicated by the absence of a pronounced plastic plateau and the progressive softening following peak load. From a design perspective, these findings highlight the necessity of adopting well-defined allowable stress criteria and appropriate safety factors when FRP components are subjected to bending loads, in order to ensure safe operation within the serviceability and damage-free domain.

### 3.3. Internal Pressure Behavior and Stress Determination

The internal pressure behavior of the FRP pipe segments was investigated using electrical resistance strain gauges bonded to the external surface of the pipe in both axial and circumferential directions. This instrumentation strategy enabled direct monitoring of strain development under pressurization and allowed experimental stresses to be determined based on the strain–stress relationships introduced previously in Equations (1)–(4). The selected internal pressure levels (0, 15, 20, 26, and 31 bar) were chosen to represent typical operational, elevated, and near-limit service conditions for industrial FRP piping systems. Pressure levels of 15 and 20 bar correspond to normal operating conditions, while 26 bar represents an elevated service regime. The maximum pressure of 31 bar was selected to approach the upper elastic–damage transition without inducing catastrophic failure, enabling detailed analysis of strain evolution and damage initiation mechanisms.

**Figure 4:**
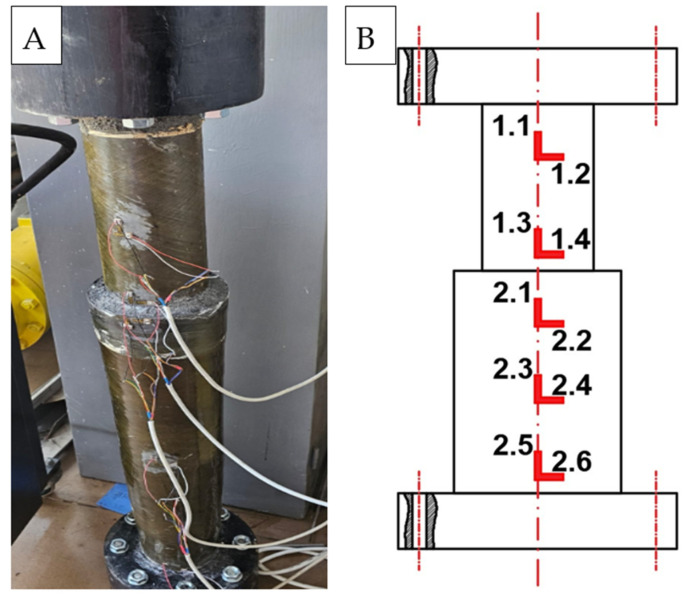
(**A**) Placement of strain gauges on the pipe, (**B**) Schematic of strain gauge locations.

Axial strain gauges were installed at locations L1.1, L1.3, L2.1, L2.3, and L2.5, while circumferential (hoop) strain gauges were positioned at locations L1.2, L1.4, L2.2, L2.4, and L2.6, as illustrated in Figure 4. This configuration ensured representative coverage of the pipe section and enabled verification of stress distribution consistency along both principal material directions.

Internal pressure tests performed on instrumented FRP pipe specimens revealed a clear pressure-dependent strain response, in good agreement with the expected mechanical behavior of filament-wound composite pipes reported in the literature [22,27].

At zero internal pressure, the measured strains are practically negligible, confirming the absence of residual stresses or measurement bias. At an internal pressure of 15 bar, moderate axial and circumferential strains are recorded, corresponding to a linear elastic response of the laminate. As the pressure increases to 20–26 bar, the measured strains exhibit an approximately proportional increase with pressure, indicating that the pipe operates within the elastic service domain defined in the previous tensile and bending analyses.

At pressure levels up to 20 bar, corresponding to normal operating conditions, the pipe exhibits a predominantly linear elastic response, characterized by proportional increases in axial and circumferential strains and stable load transfer within the laminate structure. In the intermediate pressure range between 20 and 26 bar, a gradual deviation from linearity becomes evident, indicating the initiation of microstructural damage mechanisms such as matrix microcracking and localized fiber–matrix debonding, while the global structural integrity of the pipe remains unaffected. At the maximum applied pressure of 31 bar, the pipe enters a near-limit deformation regime, where significantly higher strain gradients are recorded, particularly in the circumferential direction. This behavior reflects a pronounced hoop-dominated structural response, consistent with classical thin-walled cylinder theory and the filament-wound laminate architecture. The deformation is governed by progressive matrix cracking, interlaminar shear, and localized stiffness degradation, without sudden loss of load-carrying capacity. No catastrophic rupture was observed at this pressure level, confirming that 31 bar corresponds to the upper elastic–damage transition region rather than structural failure. This response highlights the inherent damage tolerance and structural robustness of the GFRP pipe and demonstrates stable mechanical performance within the operational safety margins prescribed by ISO 14692.

At the maximum applied pressure of 31 bar, significantly higher strain levels are recorded in both axial and circumferential directions, as summarized in Table 3, with axial strains reaching values of ε_a_ ≈ 6.99 × 10^−4^ (L1.1), 9.70 × 10^−4^ (L1.3), and 1.30 × 10^−3^ (L2.1), while circumferential strains attain ε_h_ ≈ 8.99 × 10^−4^ (L2.4), 1.20 × 10^−3^ (L2.6), and 1.60 × 10^−3^ (L2.2).

The corresponding experimentally derived stresses, calculated using Equations (3) and (4), approach the upper bound of the service stress domain or the onset of damage initiation, with axial stresses up to σ_a_^exp^ ≈ 15.3 MPa (L2.1) and circumferential stresses up to σ_h_^exp^ ≈ 18.8 MPa (L2.2)**,** without necessarily reaching catastrophic failure. This behavior is consistent with the design philosophy of FRP piping systems, where allowable stresses are defined to ensure safe operation within the elastic range and to prevent progressive damage accumulation.

The experimentally derived stress values indicate that circumferential stresses are generally higher than axial stresses, highlighting the dominant role of hoop stress in internally pressurized pipes, as evidenced by circumferential stress levels of σ_h_^exp^ ≈ 10.6–18.8 MPa compared to axial stress levels of σ_a_^exp^ ≈ 8.2–15.3 MPa depending on gauge location. This observation is in good agreement with classical thin-walled cylinder theory, as the experimental strain measurements confirm a hoop-dominated mechanical response under pressure loading, demonstrating that filament-wound GFRP laminates efficiently carry circumferential loads [48].

This behavior is also consistent with the trends previously inferred from the tensile and bending test results. Moreover, the distribution of stresses across the instrumented locations does not indicate excessive local stress concentrations, supporting the assumption of geometric and material homogeneity of the tested pipe segments.

For unrestrained pipes, the principal stresses are governed primarily by internal pressure, with hoop stress exceeding axial stress as expected. In restrained configurations, additional axial stresses may arise due to internal pressure effects, thermal expansion, and hydraulic or dynamic loads. Although the present experimental setup focuses on pressure-induced stresses, the measured strain data provide a reliable basis for extrapolating the pipe response to more complex loading scenarios addressed in design standards.

Based on the strain measurements and the experimentally derived stresses, the results confirm a good agreement between measured stress distributions and theoretical models used in FRP pipe design. This agreement validates the applicability of the constitutive relationships adopted earlier and supports the consistency of the experimental methodology with the normative framework discussed in Section 2.1.

## 4. Conclusions

The mechanical behavior of filament-wound GFRP pipes was experimentally characterized through tensile, bending, and internal pressure tests, complemented by direct strain gauge measurements. Tensile tests showed a predominantly linear elastic response up to failure, with elastic modulus values in the range of 9–12.5 GPa, followed by a sudden stress drop typical of brittle composite materials. Bending tests revealed high initial stiffness and progressive stiffness degradation after crack initiation, with failure governed by fiber rupture, matrix cracking, and interlaminar delamination.

Internal pressure testing up to 31 bar demonstrated a clear pressure-dependent strain response, with circumferential strains and stresses consistently exceeding axial values at all monitored pressure levels (0, 15, 20, 26, and 31 bar). The experimentally derived stresses confirmed a hoop-dominated behavior, in agreement with thin-walled cylinder theory and filament-wound laminate architecture. At the maximum pressure level, the measured strains approached ε_a_ ≈ 1.30 × 10^−3^ and ε_h_ ≈ 1.60 × 10^−3^, corresponding to experimentally derived stresses of σ_a_^exp^ ≈ 15.3 MPa and σ_h_^exp^ ≈ 18.8 MPa, without catastrophic failure, indicating stable elastic performance within the design range.

The experimentally determined stress distributions showed good agreement with theoretical models and with the allowable stress philosophy adopted in ISO 14692, as well as complementary ASME B31.3, ASME RTP-1, and BS 7159 design approaches. These results support the internal consistency of standardized design frameworks when coherent allowable stresses and safety factors are applied, without explicitly performing wall thickness calculations within the scope of the present study.

Overall, the results confirm that combining full-scale strain gauge measurements with standardized design equations provides a reliable basis for validating mechanical models, supporting engineering design decisions, and increasing confidence in the structural assessment of GFRP piping systems operating under internal pressure.

Future research should extend the present experimental methodology toward long-term performance aspects, including creep, fatigue, and environmental aging, as well as toward coupled experimental–numerical analyses, in order to improve damage prediction and durability assessment of filament-wound GFRP pressure pipes.

## Figures and Tables

**Table 1:** Tensile test results and mechanical properties of GFRP specimens.

No.	Strain [%]	Tensile Strength at Break [MPa]	Strain Corresponding to Linear Behavior [%]	Stress Corresponding to Linear Behavior [MPa]	Longitudinal Elastic Modulus [MPa]
1	1.18	80	0.24	30	12,500
2	1.41	84	0.28	30	10,714
3	1.24	79	0.23	27	11,739
4	1.20	66	0.24	22	9000
5	1.42	107	0.35	43	11,950
6	1.23	77	0.27	29	9650

**Table 2:** Load–displacement results from bending tests.

No	ΔL [mm]	Force [N]
1	4	1300
2	6	1400
3	4	1150
4	4	1200
5	4	1155
6	2.5	1000

**Table 3:** Experimental strain and stress results for FRP pipe segments at 31 bar.

Measurement Point	*I*_0_	*I*_a_	*I*_h_	ε_a_ [µm/m]	ε_h_ [µm/m]	ε_a_ [m/m]	ε_h_ [m/m]	σ_a_^exp^ [MPa]	σ_h_^exp^ [MPa]
L1.1	0.134	700	NA	699.90	0.00	6.99 × 10^−4^	0.00	8.240	—
L1.2	0.231	NA	1000	0.00	999.80	0.00	1.00 × 10^−3^	—	11.77
L1.3	0.183	970	NA	969.80	0.00	9.70 × 10^−4^	0.00	11.42	—
L1.4	0.267	NA	1400	0.00	1399.70	0.00	1.40 × 10^−3^	—	16.48
L2.1	0.075	1300	NA	1299.90	0.00	1.30 × 10^−3^	0.00	15.30	—
L2.2	0.308	NA	1600	0.000	1599.70	0.00	1.60 × 10^−3^	—	18.80
L2.3	0.604	700	NA	699.40	0.000	7.00 × 10^−4^	0.00	8.23	—
L2.4	0.771	NA	900	0.00	899.23	0.00	8.99 × 10^−4^	—	10.60
L2.5	0	930	NA	930.00	0.00	9.30 × 10^−4^	0.00	10.95	—
L2.6	0.625	NA	1200	0.00	1199.38	0.00	1.20 × 10^−3^	—	14.12

NA—not applicable.

## Data Availability

The original contributions presented in this study are included in the article/Supplementary Materials. Further inquiries can be directed to the corresponding authors.

## Supplementary Materials

The following supporting information can be downloaded at: https://www.mdpi.com/article/10.3390/ma19030639/s1, Figure S1: CNC High-Pressure Abrasive Waterjet Cutting Machine (YCWJ-380-X1520); Figure S2: (a) FRP pipe material prior to cutting; (b) FRP specimen after CNC water-jet cutting; Figure S3: (a) FRP specimens prepared for tensile testing; (b) tensile testing configuration during loading; Figure S4: (a) Specimens subjected to bending, (b) Bending test setup; Figure S5: Ring tensile test setup: 1—switch; 2—saddle-shaped specimen holder; 3—shear pin; (a) loading direction; (b) separation distance; (c) rounded edges; Figure S6: (a) FRP pipe segment subjected to internal pressure testing and instrumented with strain gauges; (b) schematic representation of strain gauge placement on the pipe; (C) detailed layout of axial and circumferential strain gauge positions.
