# Supplementary material for: Experimental Assessment of Stress–Strain Response in Filament-Wound GFRP Pipes Under Internal Pressure Loading
Source: Materials (Basel). 2026 Feb 6;19(3):639. doi: 10.3390/ma19030639 (PMC12898311; doi:10.3390/ma19030639)
Supplement: Supplementary file 1 [file materials-19-00639-s001.zip › materials-4140237-supplementary.pdf]

Experimental Assessment of Stress–Strain Response in Filament-Wound GFRP Pipes under Internal Pressure Loading

Costin Nicolae Ilinca, Ibrahim Naim Ramadan *, Rami Doukeh, Adrian Neacsa *, Alin DINIȚĂ, Eugen Victor Laudacescu *, Marius Gabriel Petrescu , Marius BĂDICIOIU and Ștefan Alexandru GAVRILĂ

Mechanical Engineering Department, Petroleum-Gas University of Ploiesti, 100680 Ploiesti, Romania; icostin@upg-ploiesti.ro (C.N.I); ibra@upg-ploiesti.ro (I.R.N.); rami.doukeh@upg-ploiesti.ro (R.D); adnea@upg-ploiesti.ro (A.N.); adinita@upg-ploiesti.ro (A.D.); leugen@upg-ploiesti.ro (E.V.L.), pmarius@upg-ploiesti.ro (M.G.P.); mbadicioiu@upg-ploiesti.ro (M.B.);

* Correspondence: adnea@upg-ploiesti.ro (A.N.); ibra@upg-ploiesti.ro (I.R.N.); leugen@upg-ploiesti.ro (E.V.L.)

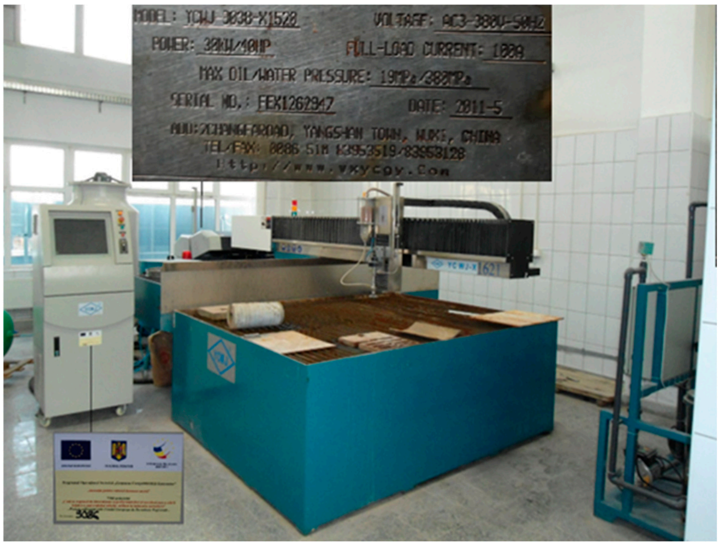

Figure S1. CNC High-Pressure Abrasive Waterjet Cutting Machine (YCWJ-380-X1520)

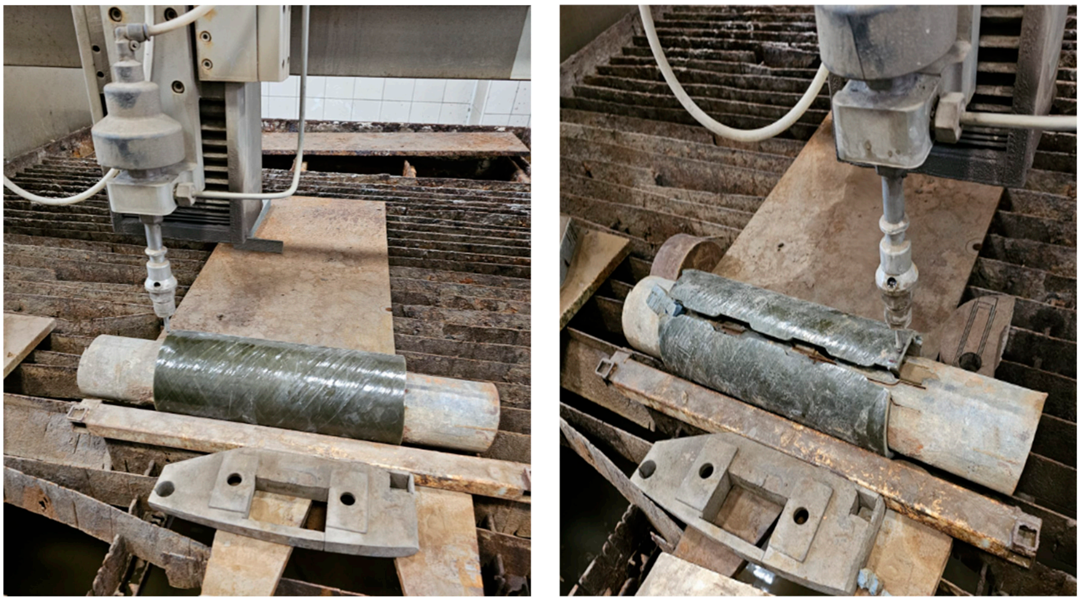

Figure S2. (**a**) FRP pipe material prior to cutting; (**b**) FRP specimen after CNC water-jet cutting.

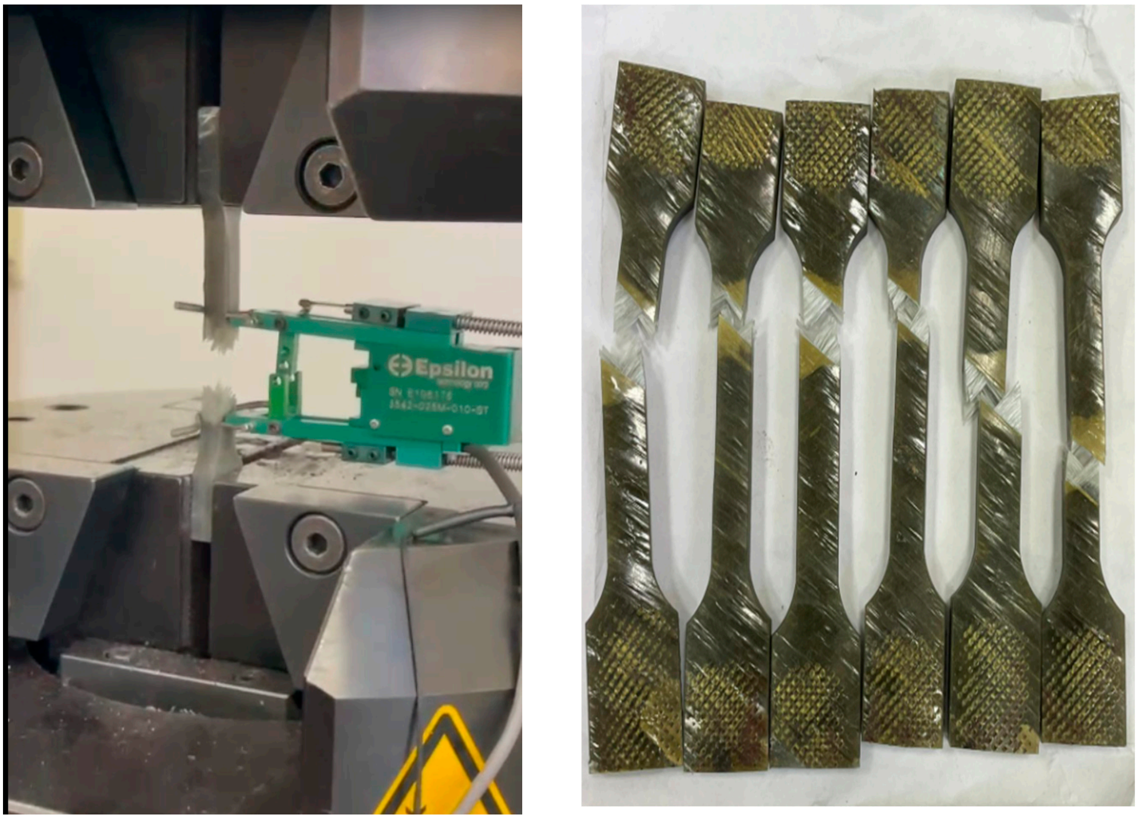

Figure S3. (**a**) FRP specimens prepared for tensile testing; (**b**) tensile testing configuration during loading.

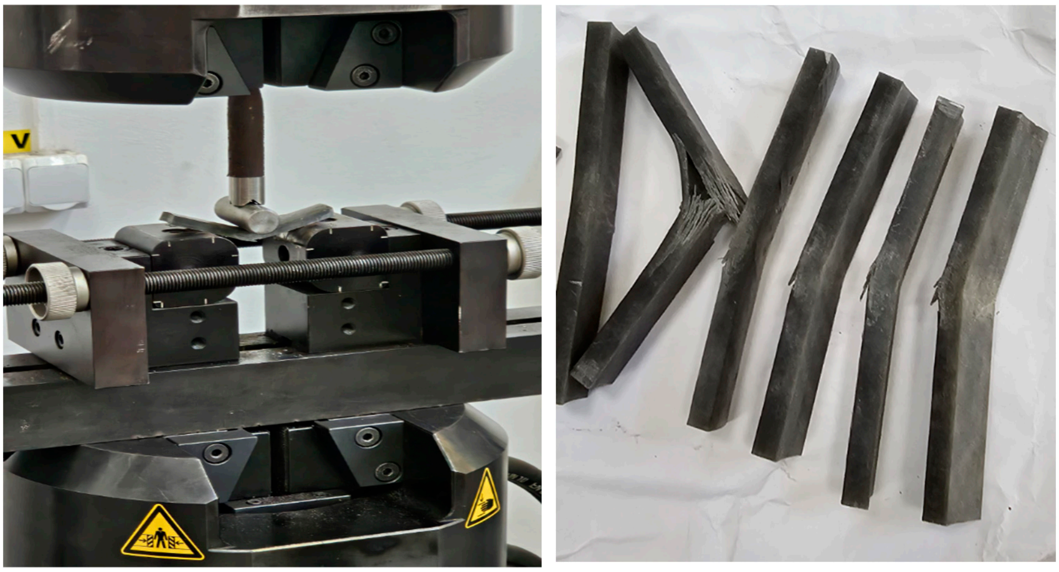

Figure S4. (**a**) Specimens subjected to bending, (**b**) Bending test setup.

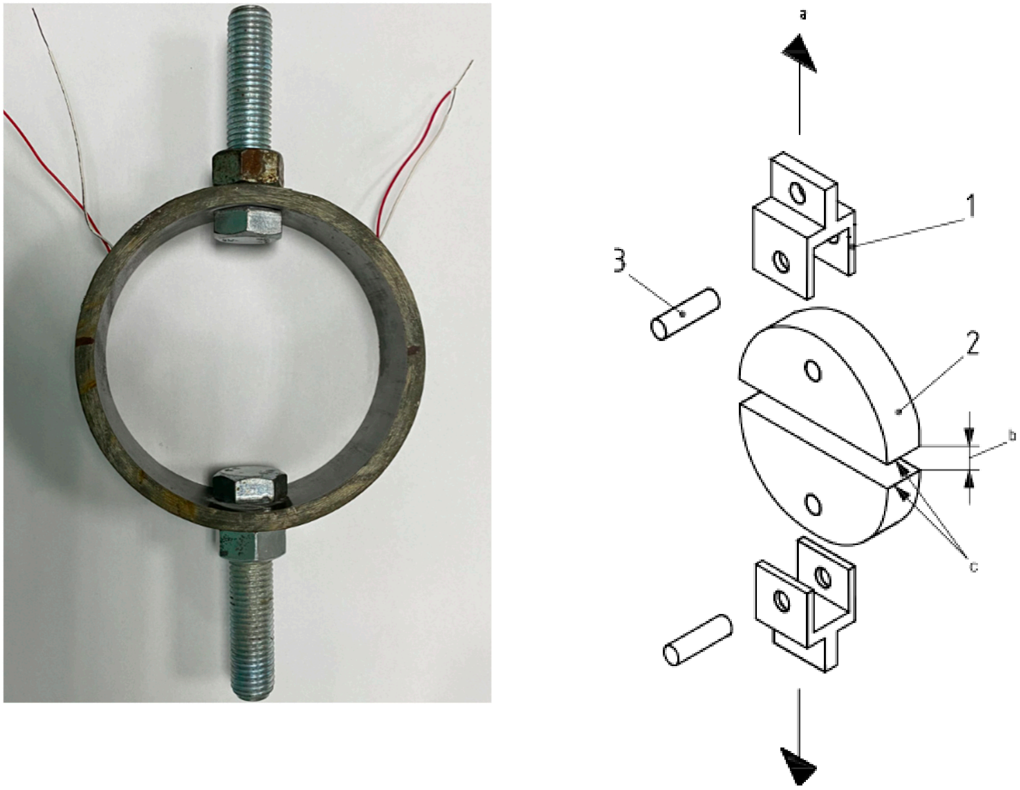

Figure S5. Ring tensile test setup: 1 – switch; 2 – saddle-shaped specimen holder; 3 – shear pin; (**a**) loading direction; (**b**) separation distance; (**c**) rounded edges.

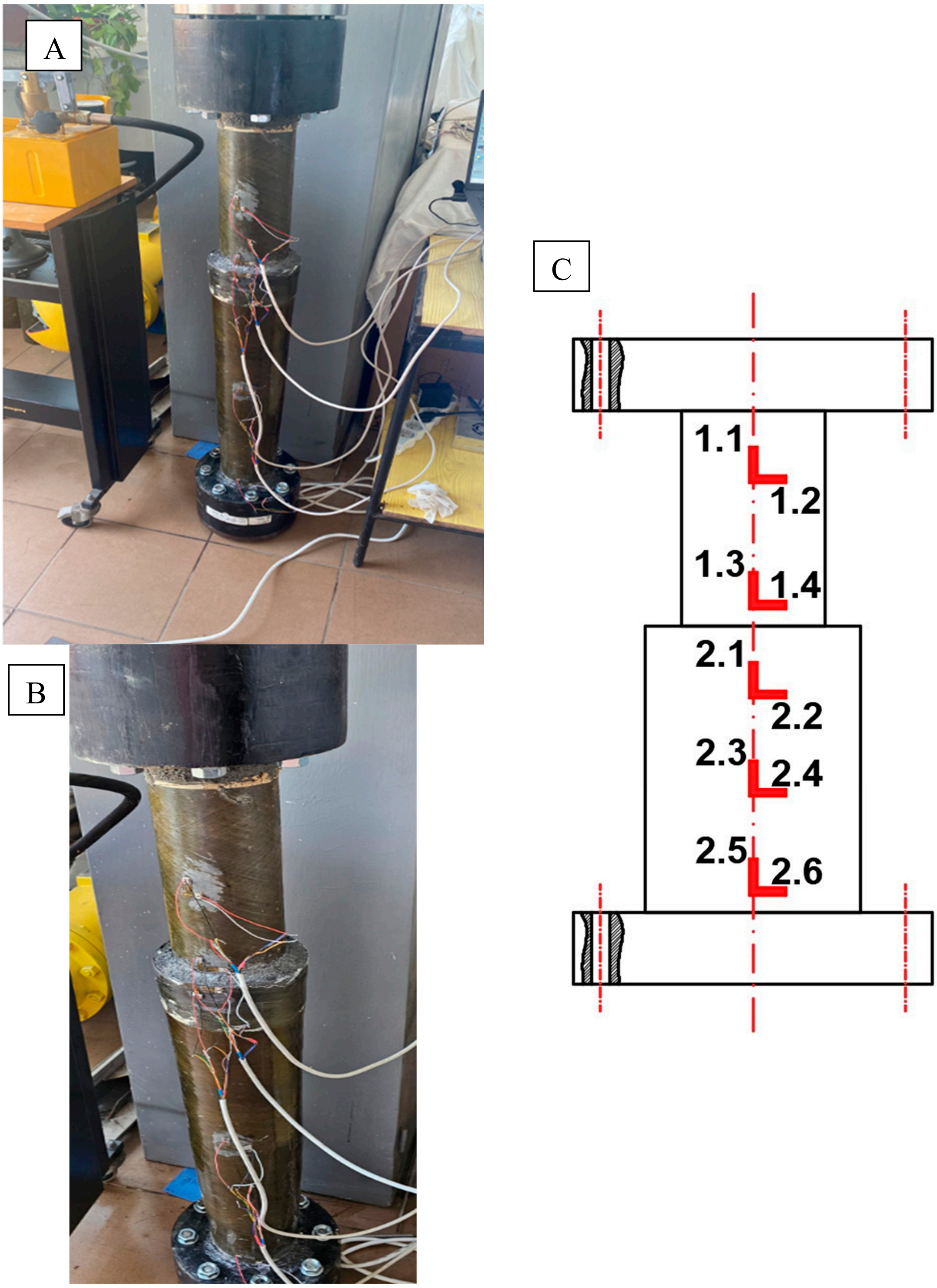

Figure S6. (**a**) FRP pipe segment subjected to internal pressure testing and instrumented with strain gauges; (**b**) schematic representation of strain gauge placement on the pipe; (**c**) detailed layout of axial and circumferential strain gauge positions.
